# Transcriptional and post-transcriptional regulation of ethylene biosynthesis by exogenous acetylsalicylic acid in kiwifruit

**DOI:** 10.1093/hr/uhac116

**Published:** 2022-05-17

**Authors:** Jian Wang, Xiao-fen Liu, Hui-qin Zhang, Andrew C Allan, Wen-qiu Wang, Xue-ren Yin

**Affiliations:** Zhejiang Provincial Key Laboratory of Horticultural Plant Integrative Biology, College of Agriculture and Biotechnology, Zhejiang University, Zijingang Campus, Hangzhou Zhejiang, 310058, China; The State Agriculture Ministry Laboratory of Horticultural Plant Growth, Development and Quality Improvement, Zhejiang University, Zijingang Campus, Hangzhou Zhejiang, 310058, China; Zhejiang Provincial Key Laboratory of Horticultural Plant Integrative Biology, College of Agriculture and Biotechnology, Zhejiang University, Zijingang Campus, Hangzhou Zhejiang, 310058, China; The State Agriculture Ministry Laboratory of Horticultural Plant Growth, Development and Quality Improvement, Zhejiang University, Zijingang Campus, Hangzhou Zhejiang, 310058, China; Institute of Horticulture, Zhejiang Academy of Agricultural Sciences, Hangzhou Zhejiang, 310021, China; New Zealand Institute for Plant & Food Research Limited, Private Bag 92169, Auckland, New Zealand; School of Biological Sciences, University of Auckland, Private Bag 92019, Auckland, New Zealand; Zhejiang Provincial Key Laboratory of Horticultural Plant Integrative Biology, College of Agriculture and Biotechnology, Zhejiang University, Zijingang Campus, Hangzhou Zhejiang, 310058, China; The State Agriculture Ministry Laboratory of Horticultural Plant Growth, Development and Quality Improvement, Zhejiang University, Zijingang Campus, Hangzhou Zhejiang, 310058, China; Zhejiang Provincial Key Laboratory of Horticultural Plant Integrative Biology, College of Agriculture and Biotechnology, Zhejiang University, Zijingang Campus, Hangzhou Zhejiang, 310058, China; The State Agriculture Ministry Laboratory of Horticultural Plant Growth, Development and Quality Improvement, Zhejiang University, Zijingang Campus, Hangzhou Zhejiang, 310058, China

## Abstract

Levels of ethylene, implicated in the induction of fruit ripening in a diverse array of plants, are influenced by genetic and environmental factors, such as other plant hormones. Among these, salicylic acid (SA) and its derivative, acetylsalicylic acid (ASA), have been demonstrated to inhibit ethylene biosynthesis in fruit, yet the underlying regulatory mechanisms remain elusive. Here, we showed that treatment with exogenous ASA dramatically reduced ethylene production, as well as activities of ACC synthase (ACS) and ACC oxidase (ACO), in kiwifruit tissues. Comparative transcriptome analysis indicated the differential expression of ethylene biosynthetic genes (*AdACS1/2* and *AdACO5*). A screen of transcription factors indicated that AdERF105L and AdWRKY29 were ASA-responsive regulators of *AdACS1/2* and *AdACO5*, respectively*.* In addition to these genes, *AdACS3* and *AdACO3* were abundantly expressed in both ASA-treated and control tissues. AdACS3 protein was phosphorylated and stabilized by AdMPK16, a mitogen-activated protein kinase, while AdACO3 activity was enhanced by AdAP, an aspartic peptidase. Exogenous ASA downregulated *AdMPK16* and *AdAP*, thereby influencing ethylene biosynthesis at a post-transcriptional level. These findings led us to propose a multidimensional system for inhibition of ethylene biosynthesis by ASA, inducing differential expression of some ethylene biosynthesis genes, as well as differential effects on protein activity on other targets.

## Introduction

Plant hormones are a series of small-molecule compounds synthesized in plants when cells are stimulated by specific signals, and are considered to be important regulators of plant growth and defense [1, 2]. A long history of phytohormonal research has helped elucidate their biosynthesis, transport, and signaling and response pathways. Ethylene is one of the earliest-discovered phytohormones, widely involved in seed germination, leaf extension, flowering induction, organ senescence, and plant responses to biotic and abiotic stresses [3]. Notably, the role of ethylene as a fruit ripening inducer makes it particularly important for horticultural crops, since altering its biosynthesis, perception, or signaling can affect the transcription and translation of many ripening-related enzymes and proteins [4, 5].

Ethylene in higher plants is derived from methionine, and the direct precursor is 1-aminocyclopropane-1-carboxylic acid (ACC) [6]. The ethylene biosynthetic pathway is comparatively simple: S-adenosyl methionine (SAM) is first converted into ACC under the catalysis of ACC synthase (ACS), and then the conversion of ACC to ethylene is achieved through ACC oxidase (ACO) [5]. Numerous studies have demonstrated that altering the transcription, translation, and protein stability of the ethylene biosynthetic enzymes ACS and ACO is the key step in manipulating ethylene production. For example, the WRKY family transcription factor AtWRKY33 directly binds to the promoter of *AtACS2/6* to induce expression in plant immunity [7]; the NAC transcription factor AtSHYG strongly transactivates the expression of *AtACO5* to regulate petiole cell expansion during root flooding [8]; and mitogen-activated protein kinases (MAPKs) MPK3/MPK6 phosphorylate two type 1 ACS isozymes (ACS2 and ACS6) to enhance their protein stability and protect them from degradation [7, 9].

Ethylene biosynthesis is affected by many environmental factors, such as temperature [10], light [11], and pathogen response [1], while multiple phytohormones participate in its regulation. For instance, exogenous gibberellin inhibits *CsACS2* and *CsACO3* to decrease ethylene production in shoot tips and regulates cucumber sexual development [12]; abscisic acid negatively modulates ethylene biosynthesis in *Arabidopsis* by transcriptionally repressing *ACS4* and *ACS8*, which is mediated by ABI4 [13]; jasmonate promotes ethylene biosynthesis and fruit ripening in apple by inducing a jasmonate signaling pathway transcription factor, MdMYC2, to activate the expression of *MdACO1* and *MdACS1* [14]; and cytokinin and brassinosteroid enhance the stability of type 2 ACS proteins to elevate ethylene biosynthesis in etiolated seedlings [15]. These studies have built on individual components to give new understanding of multi-phytohormone co-regulation in plants.

Salicylic acid (SA) is a key plant hormone required for inducing systemic acquired resistance and establishing resistance to many pathogens; it exists mainly in the form of free SA, SA 2-*O*-β-d-glucoside, and methyl salicylate [16]. The SA biosynthetic pathway has recently been clarified, after the identification of three pivotal genes, *SALICYLIC ACID INDUCTION DEFICIENT 2*, *ENHANCED DISEASE SUSCEPTIBILITY 5*, and *AVRPPHB SUSCEPTIBLE 3*, revealing the conversion from chorismate to SA in *Arabidopsis* [17, 18]. Ethylene and SA are known to synergistically accelerate plant leaf senescence and effectively coordinate plant responses to pathogens [19, 20]; however, there is also antagonism between them [21]. Previous studies showed that the ethylene signaling components EIN3 and EIL1 reduced SA production by negatively regulating the transcription of *SID2*, which encodes isochorismate synthase, required for the SA biosynthesis pathway [22]. Moreover, SA and its derivatives [methyl salicylate and acetylsalicylic acid (ASA)] have been demonstrated to affect ethylene biosynthesis and the ripening process in various fruit crops [23]. Leslie and Romani first detected decreasing ethylene production in SA-treated pear cell suspension cultures [24], which was further reported in other plants, e.g. rice [25], kiwifruit [26], and mung bean [27]. Subsequent industrial and/or research applications indicated that SA-mediated ethylene reduction is commonly associated with fruit quality, including color, softening, and disease resistance [28]. However, after research over the last three decades, the underlying regulatory mechanisms of inhibition of ethylene biosynthesis by SA have not been well elucidated.

Kiwifruit (*Actinidia* spp.) is an ideal research plant for ethylene biosynthesis since it produces large amounts of ethylene and is extremely sensitive to exogenous ethylene [29]. ASA, also known as aspirin, is a common derivative of SA, and has been used for relieving pain and fever for centuries [30]. Exogenous ASA is easily converted to SA in plants and participates in several physiological and biochemical processes. Moreover, applications of ASA in horticultural crops showed effects similar to those of SA, such as delaying fruit ripening, alleviating chilling injury, and reducing ethylene production [26, 31]. Our previous study indicated 1.0 mM ASA could delay softening of intact kiwifruit and inhibit ethylene production in kiwifruit flesh disks [23]; however, the molecular basis remains unclear. In this research, we showed that ASA strongly inhibited ethylene production as well as enzyme activities of ACS and ACO, which are considered to be encoded by five ethylene biosynthetic genes: *AdACS1*/*2*/*3* and *AdACO3*/*5*. Dual-luciferase assays and electrophoretic mobility shift assays (EMSA) indicated that AdERF105L and AdWRKY29 were ASA-responsive transcriptional regulators of *AdACS1*/*2* and *AdACO5*, respectively. In addition, an aspartic peptidase (AP) protein, AdAP, was identified through co-immunoprecipitation–mass spectrometry (CoIP–MS), which can enhance the catalytic activity of AdACO3 protein, and we verified an MAPK, AdMPK16, which stabilizes AdACS3. Both of these were downregulated by ASA treatment, resulting in reduction of the enzyme activity of ACS and ACO. In conclusion, these results provide a multidimensional understanding of ethylene biosynthesis and ASA–ethylene antagonism.

## Results

### Physiological and biochemical basis of inhibition of ethylene biosynthesis by acetylsalicylic acid

**Figure 1:**
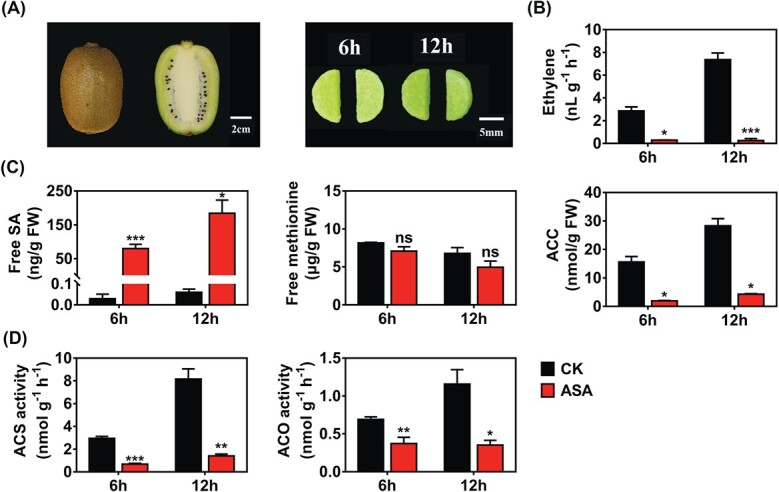
Effects of ASA treatment on kiwifruit disks. (A) Fruits were processed into flesh disks with a diameter of 1 cm and a thickness of 2 mm and then cut into two equal parts for 0.5 mM ASA treatments and control. Fruit disks were incubated at 28°C for 6 and 12 hours. Ethylene production (B), ethylene precursor (ACC), free methionine and free SA contents (C), and ethylene biosynthetic enzyme (ACS and ACO) activities (D) of kiwifruit disks. CK, control. Error bars for free SA contents indicate standard errors from two replicates; all others were from three replicates. Statistical analysis was performed using two-tailed Student’s *t*-tests. ^*^P < .1; ^**^P < .01; ^***^P < .001; ns, not significant.

‘Hayward’ kiwifruit, with firmness of 62.45 N, was processed into flesh disks (with a diameter of 1 cm and a thickness of 2 mm). Each disk was cut into two equal parts for ASA treatment and control to guarantee uniformity (Fig. 1A). A pre-experiment was conducted by determining ethylene production in kiwifruit disks under acidic and neutral conditions, and the result indicated that acidity had no influence on ethylene biosynthesis (Supplementary Data Fig. S1). As shown in Fig. 1B, ASA treatment dramatically inhibited ethylene production from 2.86 nL g^−1^ h^−1^ in control to 0.29 nL g^−1^ h^−1^ in ASA treatment in the first 6 hours, and similar results were detected in 12-hour-treated disks, which showed higher inhibitory efficiency (~30-fold). Exogenous ASA treatment triggered the accumulation of free SA (the hydrolysate of ASA), which increased from 0.029 to 79.80 ng/g at 6 hours of treatment and from 0.0599 to 183.98 ng/g at 12 hours of treatment (Fig. 1C).

The content of the direct ethylene precursor ACC was significantly lower in ASA-treated disks (1.95 and 4.32 nmol/g at 6 and 12 hours, respectively) than the control (15.60 and 28.31 nmol/g at 6 and 12 hours, respectively) (Fig. 1C). In contrast, the content of methionine, the precursor of ethylene, showed no significant difference between the ASA-treated and control disks, indicating that ethylene decrease was irrelevant to methionine content (Fig. 1C). The activities of ethylene biosynthetic enzymes (ACS and ACO) had similar trends to ACC content, and were also inhibited by ASA treatment (Fig. 1D). We propose that downregulation of ACC metabolism, mediated by ACS and ACO protein activity, was the leading cause of ethylene reduction.

### Transcriptomic analysis predicted differentially expressed ACS/ACO members and transcription factors

**Figure 2:**
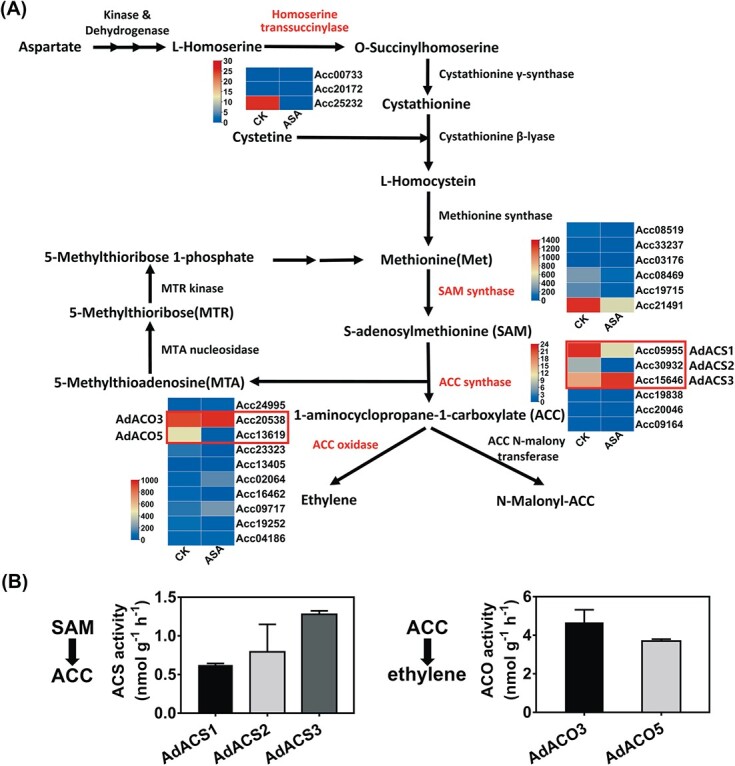
Effects of ASA treatment on expression of ethylene biosynthetic pathway genes and recombinant protein activities of five candidate genes. (A) Fruit disks were treated with 0.5 mM ASA or control (CK) at 28°C for 6 hours. The comparisons were made with three replicates at each point. The enzyme names in red are differentially expressed gene families in transcriptome data; enzyme names in black are gene families not responding to ASA. Five candidate ethylene biosynthetic genes—*AdACS1*/*2*/*3* (*Achn364251*/*Acc05955*, *Achn339101*/*Acc30932* and *Achn189421*/*Acc15646*) and *AdACO3*/*5* (*Achn326461*/*Acc20538* and *Achn157111*/*Acc13619*), highlighted by red boxes—were used for further investigation. (B) Recombinant protein activities of five candidate ethylene biosynthetic structural genes presented in the form of ethylene production. Error bars indicate standard errors from three replicates.

To investigate the key genes contributing to ethylene metabolism, RNA sequencing was carried out to select differentially expressed genes (DEGs). Six mRNA libraries were constructed, including control (6 hours) and ASA treatment (6 hours), and three biological replicates were performed. Thirty-nine Yang cycle genes and ethylene biosynthetic pathway genes were isolated by transcriptome annotation, and only four enzyme families responded to ASA treatment (Fig. 2A). The physiological and biochemical analyses (Fig. 1) guided the focus on coding genes to ACC-related enzymes (ACS and ACO). Within the ACS family, only three members were abundant in kiwifruit disks. *AdACS1/2* (*Achn364251/Acc05955* and *Achn339101*/*Acc30932*) were downregulated by ASA treatment, while *AdACS3* (*Achn189421*/*Acc15646*) showed no significant change (Fig. 2A; Supplementary Data Fig. S2A). For the ACO family, *AdACO5* (*Achn157111*/*Acc13619*) and *AdACO3* (*Achn326461*/*Acc20538*) were more highly expressed than the other members, whereas only *AdACO5* was significantly suppressed (Fig. 2A; Supplementary Data Fig. S2B). AdACS1/2/3 and AdACO3/5 recombinant proteins were purified through *Escherichia coli* strain BL21, and *in vitro* protein activity assays indicated that all recombinant proteins had catalytic activities (Supplementary Data Fig. S3; Fig. 2B). Taken together, these results indicated that five ethylene biosynthetic genes were responsible for ethylene biosynthesis in kiwifruit. The changes in transcript levels of *AdACS1*/*2* and *AdACO5* could explain part of the inhibitory effect of ASA on ethylene. Like the tremendous inhibitory efficiency of ASA on ethylene, the abundance of expression of *AdACS3* and *AdACO3* in both treated and control tissue suggests additional post-transcriptional regulation by ASA.

The dramatic decrease of *AdACS1*/*2* and *AdACO5* transcripts could likely be caused by transcription regulation. RNA-seq results showed 5059 DEGs, including 2153 upregulated and 2906 downregulated genes [fold change ≥2, false discovery rate (FDR) <.01]. These DEGs were classified into 36 groups based on two dimensions: fragments per kilobase of transcript per million fragments mapped (FPKM) and absolute fold change (Supplementary Data Fig. S4A). Two thresholds were set: (i) FPKM >200 and fold change >5 (highly expressed DEGs); and (ii) FPKM >30 and fold change >10 (highly differential DEGs). Based on these two selection criteria, 201 DEGs were further filtered (Supplementary Data Fig. S4B; Supplementary Data Table S3). Based on gene function annotation, 201 candidate DEGs were divided into seven groups, and 15 differentially expressed transcription factors (DETFs) were identified among them. These DETFs belonged to 10 family groups, including ERFs, zinc finger proteins, and WRKYs (Supplementary Data Fig. S4C).

### Transcriptional regulation of *AdACS1/2* and *AdACO5* promoters

**Figure 3:**
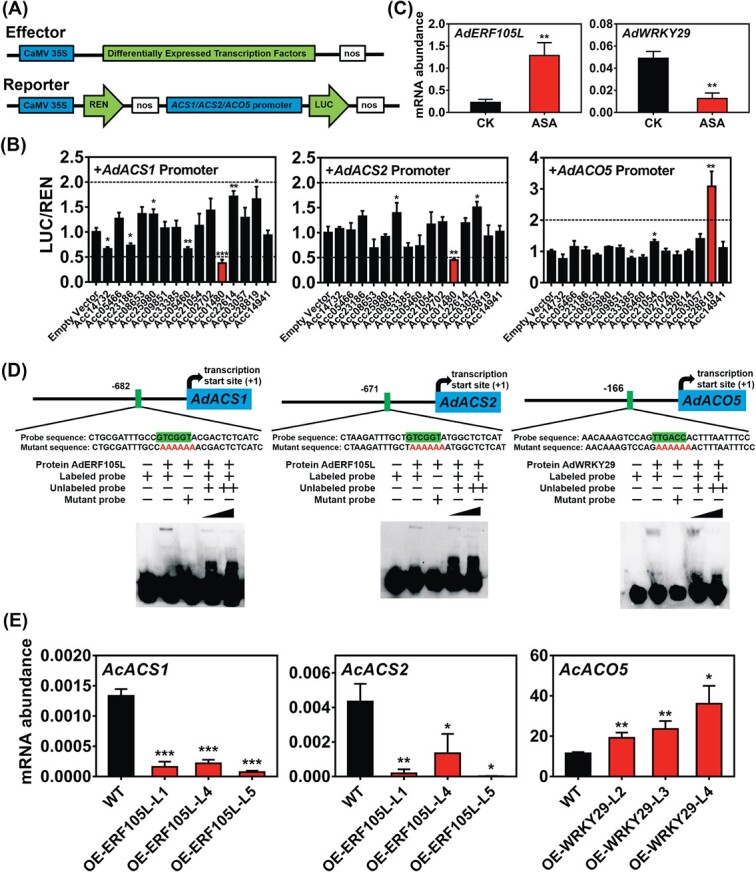
Regulatory effects of DETFs on promoters of ASA-suppressed ethylene biosynthetic genes and analysis of binding ability. (A) Schematic diagram of dual-luciferase assay. The DETFs were inserted into effector vector (SK) and the promoters were constructed into reporter vector (LUC). (B) Effects of 15 DETFs on promoters of *AdACS1*/*2* and *AdACO5*. The LUC/REN ratio of the empty vector plus promoter was set as 1. (C) Expression of two effective DETFs, *AdERF105L* (*Achn249781*/*Acc01480*) and *AdWRKY29* (*Achn132821*/*Acc28819*), in control (CK) and ASA-treated kiwifruit disks. Gene expression was analyzed by RT–qPCR and mRNA abundance was expressed relative to that of *AdACT*. (D) EMSA showed interactions between AdERF105L and *AdACS1*/*2* promoter, and between AdWRKY29 and *AdACO5* promoter. The probe sequence used for EMSA is shown and the core binding sequences are highlighted in green. Mutant probes were core binding sequence replaced with (AAAAAA), shown in red. Labeled probes and mutant probes were 3′-biotin-labeled; the unlabeled probe was a competitor; + and ++ represent 20- and 500-fold concentrations of the labeled probe, respectively. (E) Expression of *AcACS1*/*2* in wild-type (WT) and *AdERF105L*-overexpressing transgenic kiwifruit plants (lines 1, 4, and 5) and expression of *AcACO5* in WT and *AdWRKY29* overexpressing transgenic kiwifruit plants (lines 2, 3, and 4). Error bars in (B) indicate standard errors from five replicates; Error bars in (C) and (E) indicated standard errors from three replicates. Statistical analysis was performed using two-tailed Student’s *t*-tests. ^*^P < .1; ^**^P < .01; ^***^P < .001.

Fifteen DETFs were inserted into an SK vector as effectors. Meanwhile, the promoters of *AdACS1/2* and *AdACO5* were constructed into a luciferase (LUC) vector as reporters (Fig. 3A). Dual-luciferase assays were performed to determine the potential regulatory effects, and the results showed that AdWRKY29 has a significant *trans*-activation effect (*Achn132821*/*Acc28819*) on the *AdACO5* promoter. Besides, AdERF105L (*Achn249781* and *Acc01480*) repressed the *AdACS1*/*2* promoter 0.38- and 0.45-fold, respectively (Fig. 3B). RT–qPCR results indicated that *AdWRKY29* and *AdERF105L* were suppressed and induced by ASA, respectively (Fig. 3C).

**Figure 4:**
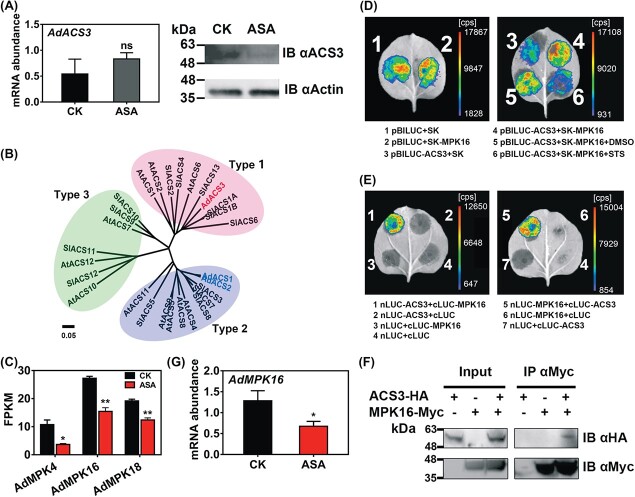
Effects of AdMPK16 on regulation of phosphorylation level and protein stability of AdACS3. (A) Gene expression and protein level of AdACS3 in control (CK) and ASA-treated kiwifruit disks, analyzed by RT–qPCR and IB, respectively. mRNA abundance was expressed relative to *AdACT*. Protein level was confirmed by IB using ACS3-specific antibody. IB with β-actin antibody indicates similar loading. (B) Phylogenetic tree analysis of ACSs in kiwifruit, *Arabidopsis*, and tomato. ACSs are divided into three types based on their C-terminus specificities. (C) FPKM value of MPKs that were suppressed by ASA treatment. A total of 18 MPKs were found in the kiwifruit genome database and three of them (*AdMPK4*/*16*/*18*) were downregulated by ASA treatment. (D) Firefly luciferase (LUC) imaging assays indicate that AdMPK16 (*Achn252431*/*Acc01953*) stabilizes AdACS3 protein and prevents it from degradation. The strength of LUC signal (shown as different colors) indicates the protein stability of AdACS3. Empty pBILUC plus SK or AdMPK16 are negative controls, showing no influence of AdMPK16 on LUC protein (1, 2). STS, staurosporine, inhibitor of protein kinase; DMSO, dimethyl sulfoxide, solvent of STS, used as negative control. (E) Interactions between AdACS3 and AdMPK16 shown by firefly LCI assays. Colored regions indicate protein–protein interactions (1, 5). Empty nLUC or cLUC plus corresponding AdAP and AdACO3 were negative controls (2–4, 6, 7). (F) CoIP assay showing the interaction between AdMPK16 and AdACS3. Total protein from tobacco leaves expressing MPK16-Myc was immunoprecipitated (IP) with anti-Myc antibody-conjugated agarose beads. The IP and input samples were detected by IB using HA and Myc antibodies. (G) Gene expression of *AdMPK16* in CK and ASA-treated kiwifruit disks, analyzed by RT–qPCR. Error bars in (A), (C) and (G) indicate standard errors from three replicates. Statistical analysis was performed using two-tailed Student’s *t*-tests. ^*^P < .1; ^**^P < .01; ns, not significant.

To further investigate the physical interactions between DETFs and promoters, recombinant proteins of AdWRKY29 and AdERF105L were purified, and EMSA was conducted. The W-box element (C/T)TGAC(C/T) is the core *cis*-element for WRKY members [32]. There were three W-box motifs in the *AdACO5* promoter (Supplementary Data Table S4), and only the region located between −166 and −161 bp showed a binding band. When mutant probe, where the core binding sequence was replaced with AAAAAA, or unlabeled probe was added, the binding band disappeared or was reduced, respectively (Fig. 3D). ERF family proteins have been reported to bind G-box [33] element AGCCGCC or DRE [34] element (A/G)CCGAC. One G-box and two DRE motifs were found in the promoters of both *AdACS1* and *AdACS2* (Supplementary Data Table S4), with a binding region found to be located −682 to −677 bp in *AdACS1* promoter (DRE) and − 671 to −666 bp in *AdACS2* promoter (DRE), respectively. The effects of mutant probes and unlabeled probes verified this binding (Fig. 3D). Besides, three *AdERF105L*-overexpressing transgenic lines (lines 1, 4, and 5) and three *AdWRKY29*-overexpressing transgenic lines (lines 2, 3, and 4) of kiwifruit plants confirmed the regulatory effects of these two DETFs *in vivo*, via repressing or activating the expression of downstream ethylene biosynthetic genes (Supplementary Data Fig. S5; Fig. 3E).

### Acetylsalicylic acid influenced AdACS3 stability via mitogen-activated protein kinase

Although recombinant AdACS3 protein had high ACS activity (Fig. 2B), transcriptome data and RT–qPCR showed that expression of *AdACS3* did not respond to ASA treatment (Fig. 4A; Supplementary Data Fig. S2A). To determine the protein abundance of AdACS3, immunoblotting analysis (IB) was carried out by using an AdACS3-specific antibody. The results revealed that the ACS3 protein level was lower in ASA-treated disks (Fig. 4A). To investigate whether protein reduction of AdACS3 was mediated by the ubiquitin–proteasome system, the ubiquitination inhibitor MG132 was applied to kiwifruit disks together with ASA. The results showed that MG132 did not influence the protein level of AdACS3, and ASA-dependent ethylene decrease was not affected either (Supplementary Data Fig. S6).

**Figure 5:**
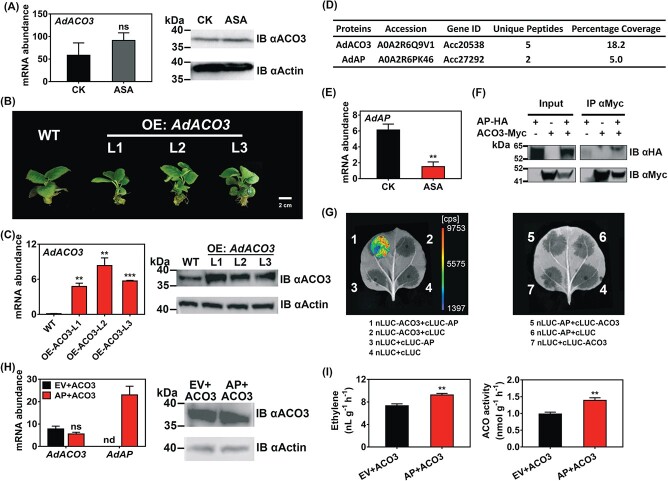
Effects of AdAP on regulation of protein activity of AdACO3. (A) Gene expression and protein level of AdACO3 in control (CK) and ASA-treated kiwifruit disks, analyzed by RT–qPCR and IB, respectively. mRNA abundance was expressed relative to *AdACT*. Protein level was confirmed by IB using ACO3-specific antibody. IB with β-actin antibody indicates similar loading. (B) Two-month-old transgenic kiwifruit (*A. chinensis* cv. ‘Donghong’) plants were used to extract total proteins to conduct CoIP. (C) Gene expression and protein abundance of AdACO3 in wild-type (WT) and *AdACO3*-overexpressing (OE:*AdACO3*) transgenic kiwifruit plants. (D) AdAP protein was identified in ACO3 complexes by LC–MS/MS analysis. Beads conjugated with AdACO3-specific antibody were used for immunoprecipitation (IP) of proteins from extracts of transgenic kiwifruit plants and IP with rabbit normal IgG was used as a control. ‘Accession’ indicates protein serial number in the FASTA database. ‘Unique Peptides’ represents the number of distinct peptide sequences that match the indicated protein. ‘Percentage Coverage’ shows the percentage of the protein sequence covered by identified peptides. (E) Gene expression of *AdAP* (*Achn106461*/*Acc27292*) in CK and ASA-treated kiwifruit disks. (F) CoIP assay showing the interaction between AdAP and AdACO3. Total protein from tobacco leaves expressing ACO3-Myc was subjected to IP with anti-Myc antibody-conjugated agarose beads. The IP and input samples were detected by IB using HA and Myc antibodies. (G) Interactions between AdAP and AdACO3 shown by the firefly LCI assay. Colored regions indicate protein–protein interactions (1). Empty nLUC or cLUC plus corresponding AdAP and AdACO3 were negative controls (2–4, 6, 7). The absence of colored regions in 5 indicates that the interaction only occurred in one direction. (H) Transient expressing ACO3 and AP in tobacco. ACO3 and AP were mixed in a ratio of 1:1 and infiltrated into tobacco leaves. ACO3 plus empty vector was used as a control. Gene expression and protein abundance of AdACO3 in tobacco leaves were tested by RT–qPCR and IB. (I) Ethylene production and ACO activity of transient infiltrated tobacco leaves. Error bars in (A), (C), (E), (H) and (I) indicate standard errors from three replicates. Statistical analysis was performed using two-tailed Student’s *t*-tests. ^**^P < .01; ^***^P < .001; ns, not significant; nd, not detected.

Phylogenetic tree analysis indicated that AdACS3 belongs to the type 1 ACS subfamily (Fig. 4B), which contains target sites for MAPK at the C terminus [5]. To explore the potential phosphorylation of AdACS3, a total of 18 MAPK genes were obtained from the kiwifruit genome database, and three of them (*AdMPK4/16/18*) were downregulated by ASA treatment (Fig. 4C). The firefly luciferase imaging assay showed that AdMPK16 (*Achn252431*/*Acc01953*) could stabilize the AdACS3 protein and prevent degradation. Meanwhile, it had no influence on empty pBILUC vector (Fig. 4D). When staurosporine (inhibitor of protein kinase) was added, the strength of the LUC signal became extremely low, indicating that AdACS3 protein was phosphorylated (Fig. 4D). However, similar effects could not be observed in AdMPK4/18-infiltrated leaves (Supplementary Data Fig. S7). In addition, firefly luciferase complementation imaging (LCI) and CoIP assays indicated that there was a protein–protein interaction between AdMPK16 and AdACS3 protein *in vivo* (Fig. 4E and F). Furthermore, RT–qPCR also confirmed that *AdMPK16* was inhibited by ASA treatment (Fig. 4G). These results suggest that exogenous ASA treatment downregulates the expression of *AdMPK16*, which keeps the protein stability and protein abundance of AdACS3, and hence partially contributes to the sharp decrease in ACS enzyme activity.

### Acetylsalicylic acid influenced AdACO3 protein activity via aspartic peptidase

Unlike AdACS3, gene expression and protein abundance of *AdACO3* were not affected by ASA treatment (Fig. 5A). To find any other potential regulators of AdACO3, a CoIP–MS assay was performed using *AdACO3*-overexpressing kiwifruit plants to identify proteins with which AdACO3 forms complexes (Fig. 5B and C). Beads conjugated with AdACO3-specific antibody were used to immunoprecipitate (IP) protein extracts from *AdACO3*-overexpressing kiwifruit plants, with normal rabbit IgG used as a control. By removing duplicate proteins in the control, 92 unique peptides were found in immunoprecipitates from the extracts by LC–MS/MS analysis, including AdACO3 itself (Supplementary Data Table S5). Combined with transcriptome data, a highly expressed and ASA-responsive gene, *AdAP* (*Achn106461*/*Acc27292*), was found (Fig. 5D and E; Supplementary Data Table S5). CoIP and LCI assays confirmed the interaction between AdACO3 and AdAP *in vivo* (Fig. 5F and G). In addition, the regulatory effects of AdAP on AdACO3 protein activity were detected via transient assays. RT–qPCR and IB results showed that AdAP did not influence gene expression or protein abundance of AdACO3 (Fig. 5H), but enhanced the enzyme activity of AdACO3 together with an increase in ethylene production compared with the control (Fig. 5I). These results indicated that AdAP increased ethylene production via enhancing the protein activity of AdACO3, while exogenous ASA treatment inhibited this process, eventually leading to ethylene decrease.

### Inhibition of ethylene biosynthesis by acetylsalicylic acid in various fruits

**Figure 6:**
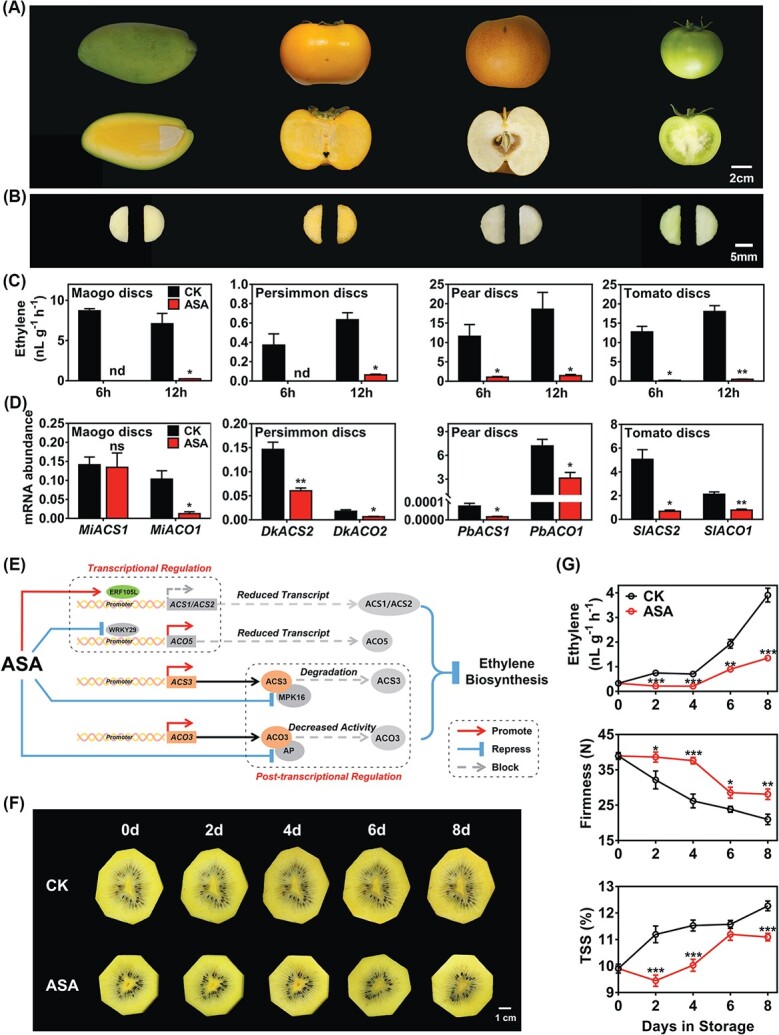
Verification of regulatory effects of ASA on ethylene biosynthesis in diverse fruit tissue disks and fresh-cut kiwifruit. Four different fruits were harvested at commercial maturity (A) and processed into disks (B). Ethylene production (C) and expression of pivotal ACSs and ACOs (D) were measured. Gene expression was analyzed by RT–qPCR in disks treated for 6 hours and mRNA abundance was expressed relative to *MiACT*, *DkACT*, *PbACT*, and *SlACT*. (E) Proposed model for the mechanism by which exogenous ASA inhibits ethylene biosynthesis. AdERF105L is an ASA-induced transcriptional repressor of *AdACS1*/*2* and AdWRKY29 is an ASA-suppressed transcriptional activator of *AdACO5*. Both of them suppress expression of ethylene biosynthetic genes through promoter binding. Meanwhile, ASA reduces expression of *AdMPK16* and *AdAP*. *AdMPK16* encodes a protein kinase that stabilizes AdACS3 protein by increasing phosphorylation level, and *AdAP* encodes a proteolytic enzyme that enhances protein activity of AdACO3. Both of them inhibit ethylene biosynthesis at a post-transcriptional level. (F) Peeled kiwifruits were processed into slices and immersed in 1 mM ASA or water (CK) for 10 min. After drying with filter paper, the slices were stored at 4°C for further physiological analysis. (G) Ethylene production, fruit firmness, and total soluble solids (TSS) of fresh-cut kiwifruit slices during cold storage. Error bars in (C) and (D) indicate standard errors from three replicates. Error bars for ethylene production indicate standard errors from three replicates; firmness and TSS were from 12 replicates. Statistical analysis was performed using two-tailed Student’s *t*-tests. ^*^P < .1; ^**^P < .01; ^***^P < .001; nd, not detected.

Four different fruits (mango, persimmon, pear, and tomato) were used to test the wide effect of ASA on ethylene biosynthesis. All fruits used in this experiment were harvested at commercial maturity (Fig. 6A), and all of them were processed into disks (Fig. 6B) and treated with ASA for 6 or 12 hours. The results showed that exogenous ASA dramatically inhibited ethylene production in these fruit disks and ethylene in ASA-treated mango and persimmon disks was undetectable at 6 hours of treatment (Fig. 6C). We obtained several pivotal ACS and ACO genes in these four species, including *MiACS1*/*MiACO1* (GenBank no. AF170705/AJ505610), *DkACS2*/*DkACO2* [35], *PbACS1*/*PbACO1* (GenBank no. AB265794/AB265797), and *SlACS2*/*SlACO1* (GenBank no. NM001247249/NM001247095), and analyzed their gene expression. Except for *MiACS1*, all other genes were downregulated by ASA treatment (Fig. 6D), which indicated a similar response to ASA in ethylene production in diverse fruit species. In addition, the effect of ASA injection on intact mature green tomato fruit was shown by a delayed color change around the injection orifices (Supplementary Data Fig. S8).

Taking the results together, we elucidated the molecular basis of inhibition of exogenous ASA on ethylene biosynthesis (Fig. 6E). Based on the molecular mechanisms, we expanded and applied ASA to fresh-cut fruit in the 2021 season. As expected, exogenous ASA treatment inhibited ethylene production to maintain fruit appearance and quality (Fig. 6F and G). Moreover, ASA-treated kiwifruit slices retained higher fruit firmness and lower total soluble solids (TSS) (Fig. 6G), which are two fundamental physiological indicators of kiwifruit ripening [29].

## Discussion

Kiwifruit is a typical climacteric fruit and is extremely sensitive to exogenous ethylene. Recent studies have progressively identified biosynthetic genes, transcription factors and microRNAs involved in ethylene response in kiwifruit, providing a wider perspective to understand kiwifruit ripening [29, 36–38]. Multiple environmental factors and plant hormones have been confirmed to regulate kiwifruit ethylene biosynthesis, such as exogenous ethylene [29], methyl jasmonate [37], and ozone [39]. Previous studies indicated the correlation between exogenous ASA and kiwifruit softening and ripening [23, 26]. To elucidate the regulatory mechanism, this study put emphasis on ethylene biosynthesis and we chose kiwifruit flesh disks as the major material. We showed that ASA, an important derivative of SA, inhibited ethylene production in kiwifruit flesh disks, and similar inhibitory effects could be observed in fruit disks of other crops (Figs 1B and 6C), as well as intact tomato fruit and fresh-cut kiwifruit (Fig. 6F; Supplementary Data Fig. S8). Despite the previous reports on the inhibitory effect of SA and/or ASA on ethylene production [24, 25], investigation of the underlying mechanisms has mainly focused on the inhibition of gene expression and enzyme activity [23, 26]. Consistent with previous research, the present findings indicated that ASA inhibition of ethylene production in kiwifruit can be considered to be obstructed at two steps, from SAM to ACC and from ACC to ethylene, as little change could be observed in the content of methionine (Fig. 1C). However, ACC content, as well as ACS/ACO activities, were significantly reduced (Fig. 1C and D).

The unbalanced status existed for decades between the physiological, biochemical and molecular basis for ASA-inhibition of ethylene production. The present results indicate the complexity of regulatory mechanisms underlying the ways in which ASA affects ethylene biosynthesis, involving both transcriptional and post-transcriptional regulation (Fig. 6E). *AdACS1*/*2* and *AdACO5* were significantly downregulated by ASA treatment, which was modulated by two transcription factors, AdERF105L and AdWRKY29. Of these, AdERF105L functioned as a transcriptional repressor of *AdACS1/2*, whereas AdWRKY29 was a transcriptional activator of *AdACO5.* WRKY is one of the largest plant-specific transcription factor families [32], some of which are rapidly induced when plants are subjected to various stresses or defense signals, including SA accumulation [40]. The regulatory effects of WRKYs on ethylene biosynthesis via the repression of the promoters of *TaACS2*/*7*/*8* have been reported previously in wheat [41]. Moreover, AtWRKY33 could directly activate the expression of *ACS2* and *ACS6* in response to pathogen invasion, and so play a key role in determining the kinetics and magnitude of ethylene induction in *Arabidopsis* [7]. The regulation of ethylene production by ERFs has also been discovered in various fruits, including apple [14] and banana [42]. Our findings indicate that inhibition of ethylene biosynthesis by ASA in kiwifruit disks was partially controlled via the transcriptional regulation of *AdACS1*/*2* and *AdACO5* expression by AdERF105L and AdWRKY29 (Fig. 6E).

As mentioned above, the complexity of regulation of ethylene production by ASA also involved post-transcriptional regulation (Fig. 6E), as the expression of *AdACS3* and *AdACO3* was not altered by ASA treatment (Figs 4A and 5A). In response to ASA treatment, AdACS3 protein levels decreased while the AdACO3 protein levels were maintained (Figs 4A and 5A). Earlier studies have demonstrated that post-transcriptional regulation of the ACS protein is vital for ethylene biosynthesis [15, 43]. The ACS family can be divided into three subfamilies [5] based on the specificity of the C terminus: type 1, with MAPK- and calcium-dependent protein kinase target sites; type 2, with calcium-dependent protein kinases and E3 ligase target sites; and type 3, without target sites. Phosphorylation of type 1 ACSs has been shown to increase ACS protein stability and thus induce ethylene production. In *Arabidopsis*, MPK3/MPK6 stabilizes ACS2/ACS6 protein *in vivo*, which greatly promotes ethylene biosynthesis when stimulated by various stresses [7, 9]. Our results indicate that the downregulated expression of *AdMPK16* by exogenous ASA treatment reduced protein stability and promoted the degradation of AdACS3 protein, a type 1 ACS (Fig. 4D and G), potentially resulting in an ethylene decrease in kiwifruit disks.

The regulatory mechanism of ASA on the activity of AdACO3, which is stable and abundant at both mRNA and protein levels, was revealed using stable transgenic kiwifruit. By analyzing proteins that co-immunoprecipitated with ACO3, we identified an AP protein that exhibited high expression and significant differences between control and ASA-treated disks (Supplementary Data Table S5). AdAP had the function of enhancing the activity of AdACO3 and subsequently increasing ethylene production (Fig. 5I). APs are a group of proteolytic enzymes with two aspartic acid residues as catalytic active sites. The majority of plant APs are active at acidic pH and contain a C-terminal domain of ~100 amino acids, named the plant-specific insert (PSI) [44]. Although the understanding of AP functions in plants is not as clear as in animals and microorganisms, typical APs have been associated with plant growth and defense, including pollen germination [45] and drought response [46]. Moreover, previous research found that *SPAP1* encodes a functional aspartic protease in sweet potato [47]. Exogenous application of SPAP1 fusion protein in potato leaves significantly accelerated ethephon-induced leaf senescence and this promotion could be eliminated by an aspartic protease inhibitor [47]. Similar effects of APs on plant senescence have also been reported in grape [48]. However, the direct correlations between APs and ethylene production have not been reported yet. In view of the critical role of ethylene in ripening and senescence, we acquired some clues to establish a connection between APs and ethylene biosynthesis. Based on our findings, it could be proposed that the formation of the AP-ACO3 complex enhances ACO3 activity to promote ethylene production. Downregulated expression of *AdAP* by ASA treatment reduced the complex and inhibited biosynthesis of ethylene. However, more experimental evidence is needed to determine whether AdAP-mediated promotion of AdACO3 activity and ethylene production is associated with the PSI domain and aspartic acid residues.

In conclusion, multiple layers regulatory mechanisms of ASA-inhibition of ethylene production are getting involved of both transcriptional (*AdACS1*/*2* and *AdACO5*) and post-transcriptional (*AdACS3 and AdACO3*) regulation, which provided the in-depth advances on this long-term existing inhibitory effects. By studying the significant inhibitory effects of ASA on ethylene production, using both simplified fruit disks and stable transgenic plants, new regulators of ethylene biosynthesis have been revealed.

## Materials and methods

### Plant material and treatments

Kiwifruit (*Actinidia* spp.) was harvested from a commercial orchard in Shaanxi Province, China, in 2018, with mean TSS of 6.68%. Fruits of uniform size and free of mechanical damage were chosen and processed into flesh disks (skin, seeds, and core tissue were excluded; disks were cut entirely from the outer fruit flesh, and were 1 cm in diameter and 2 mm thick). Each disk was cut into two equal parts for reagent treatment and control experiments. The tested group was treated with 0.5 mM ASA (the pH of the ASA solution was 3.5) while the control group was acidified to pH 3.5 with hydrochloric acid; both solutions were supplemented with 0.4 M mannitol to maintain osmotic pressure and protect the disks from rupturing or dehydration. The ASA concentration and pH were chosen as optimal from preliminary experiments: 0.5 mM ASA treatment has better inhibitory effects than the previously reported [26] 1.0 mM. The treatment time was set at 6 or 12 hours. Each group contained 300 half-disks, separated into three replicates of 100 half-disks each and collected in a 100-mL conical flask filled with 50 mL of solution. The flasks were covered with tin foil, then placed on a shaker (25 rpm) away from light and incubated at 28°C. After incubation, the disks were dried with filter paper, then 15 of them were used for ethylene measurement and the rest were frozen in liquid nitrogen and then stored at −80°C.

### Ethylene measurement

Ethylene production was measured according to our previous report [23, 29]. The dried disks were sealed in a 15-mL syringe with a rubber stopper for 1 hour. One milliliter of headspace gas was collected with a 1-mL injector from the syringe, with three replicates. The ethylene was measured with a gas chromatograph (Agilent Technologies 7890A GC System) fitted with a ProPak Q column. The temperatures of the injector, detector, and oven were 140, 230 and 100°C, respectively.

### ACC content, ACC synthase, and ACC oxidase

The content of ACC and activities of ACS and ACO were measured following our previous reported methods [37]. Approximately 2 g of frozen disks was used for ACC extraction and 1 mL of headspace gas collected from the ACC reaction system was used for ACC determination. Internal standard of ACC (0.1 mM) was added to calculate the conversion efficiency of ACC to ethylene. ACC synthase and oxidase activities were determined with 3 and 4 g of frozen disks, respectively. The ACC content and enzyme activities were analyzed by measuring ethylene production from the reaction system, using the gas chromatograph mentioned above. All the measurements were performed with three biological replicates.

### Free amino acid and free salicylic acid assays

Free amino acids were extracted from 1 g of ground disks with 8 mL of 3% (w/v) sulfosalicylic acid [38] and the mixture was placed on a shaker at 200 rpm for 1 hour at 4°C. The suspension was then centrifuged at 9000 rpm for 10 min at 4°C and clarified by passage through a 0.45 μm cellulose acetate filter. Amino acids in the supernatant were further analyzed using the ninhydrin method with an automatic amino acid analyzer (L-8900; Hitachi, Tokyo, Japan).

For free SA measurement, 0.1 g of frozen ground sample was used following the previously described protocol [49]. One milliliter of ethyl acetate spiked with 50 ng of D4-SA, used as the internal standard for SA, was added to each sample and high-speed oscillated on a digital shaking drybath overnight at 4°C. The suspension was then centrifuged at 12 000 rpm for 10 min at 4°C and supernatants were collected in fresh 2-mL tubes. Each pellet was re-extracted with 0.5 mL of ethyl acetate with internal standard and centrifuged; supernatants were combined and evaporated to dryness on a rotary evaporator at room temperature for 2 hours. Then 0.5 mL of 70% chromatographic methanol was added to dissolve and mix the residue. After centrifugation at 12 000 rpm for 10 min, the supernatants were collected in autosampler vials and then analyzed by HPLC.

### RNA extraction and RNA-seq

Total RNA and genomic DNA were extracted by the cetyltrimethylammonium bromide method [36]. RNA extracted from ASA-treated and control disks with three replicates were sent for RNA-seq. Sequencing libraries were generated using the NEBNext Ultra™ RNA Library Prep Kit for Illumina (USA) and sequenced on an Illumina Hiseq Xten platform. Clean data were obtained by removing reads containing adapter, poly-N, and low-quality reads from raw data, and were then mapped to the cv. ‘Hong Yang’ (*Actinidia chinensis*) genome database by Tophat2 tools. Quantification of gene expression levels was estimated by FPKM. Genes with fold change ≥2 and FDR <.01 were assigned as differentially expressed.

### cDNA synthesis and RT–qPCR

For cDNA synthesis, gDNA removal and first-strand cDNA synthesis were conducted using the PrimeScript™ RT Reagent Kit (Takara, Beijing, China) with 0.5 μg of total RNA for each sample. For RT–qPCR, LightCycler^®^ 480 (Roche) and LightCycler^®^ 480 SYBR Green I Master (Roche) were used. The reaction systems and procedures were performed as previously described [36]. Primers for RT–qPCR were designed by Primer3 (v.0.4.0; https://bioinfo.ut.ee/primer3-0.4.0/) and verified by melting curve analysis. Kiwifruit *actin*, mango *actin*, persimmon *actin*, pear *actin*, and tomato *actin* (GenBank no. EF063572, AF246288, AB473616, GU830958, and AB199316) were used as housekeeping genes. Primers for RT–qPCR are listed in Supplementary Data Table S1.

### Dual-luciferase assay

Full lengths of 15 selected DETFs were inserted into pGreen II 0029 62-SK vector [50], while promoters of *AdACS1*/*2* and *AdACO5* were recombined into the pGreen II 0800-LUC vector [50]. Sequences of primers for vector construction are listed in Supplementary Data Table S2. All the constructs were transferred into *Agrobacterium tumefaciens* GV3101, then cultured and dissolved in infiltration buffer (150 μM acetosyringone, 10 mM MES, and 10 mM MgCl_2_, pH 5.6), and finally adjusted to an OD_600_ of 0.75. The prepared cultures of DETFs and promoters were mixed at a ratio of 10:1, and infiltrated into tobacco leaves (*Nicotiana benthamiana*). Three days after infiltration, firefly luciferase and *Renilla* luciferase were analyzed with the Dual-Luciferase Reporter Assay System (Promega). Dual-luciferase assays were conducted with five biological replicates.

### Recombinant protein purification and electrophoretic mobility shift assay

The coding sequences of *AdWRKY29* and *AdERF105L* were recombined to pGEX-4 T-1 vector then transformed into *E. coli* strain BL21 (DE3). The full-length coding sequences of *AdACS1*/*2*/*3* and *AdACO3*/*5* were inserted into pET-32a vector and then transferred into *E. coli* strain BL21 (DE3). Recombinant proteins were induced by 1 mM IPTG (isopropyl β-d-thiogalactoside) at 16°C for 20 hours, then purified using the GST-tag Protein Purification Kit (Beyotime, Shanghai, China) or HisTALON Purification Kit (Takara, Beijing, China). Corresponding primers are listed in Supplementary Data Table S2.

The probes used for EMSA were 3′-biotin end-labeled by HuaGene (Shanghai, China) and converted to dsDNA probes by annealing complementary oligonucleotides. EMSA was conducted using a LightShift Chemiluminescent EMSA kit (Thermo Fisher Scientific). Mutant probes and competition probes (20- and 500-fold unlabeled oligonucleotides) were used to verify the binding specificity.

### Protein extraction and immunoblotting

Protein extraction was conducted using a plant protein extraction reagent (FDBIO, Hangzhou, China). Protease inhibitor mixture was added at a ratio of 1:99 to the pre-cooled extraction buffer before use. Kiwifruit samples pooled from flesh fruit disks and tissue-cultured leaves were ground into powder in liquid nitrogen, and 0.5 mL of extraction buffer was used for every 0.1 g of sample. After incubation on ice for 30 minutes, the suspension was centrifuged at 12 000 rpm for 20 minutes at 4°C, and then the supernatant was collected in a fresh tube. Protein concentration was measured with a BCA protein assay kit (FDBIO, Hangzhou, China).

Proteins extracted from tobacco or kiwifruit were separated on 12% SDS–PAGE gels and transferred to PVDF membranes (Bio-Rad) using a highly efficient wet protein transfer system (eBlot L1; GenScript, Nanjing, China). HA (hemagglutinin)-tagged or Myc-tagged fusion proteins were detected using the rabbit polyclonal HA or Myc antibody at 1:2500 dilution (HuaBio; 0906, R1208). Mouse monoclonal β-actin antibody (CWBio; CW0096M) was used at 1:2000 dilution. Mouse monoclonal His antibody (Abcam, Cambridge, UK) was used at 1:2500 dilution. After incubation with the secondary antibody, IB signals were obtained using a chemiluminescence kit (FDBIO; FD8000). To detect the protein abundance of AdACS3 and AdACO3 in kiwifruit disks, rabbit polyclonal antibodies specific for AdACS3 and AdACO3 were obtained (HuaBio, Hangzhou, China). The antibodies were made by immunizing rabbits with purified recombinant mature AdACS3 or AdACO3 protein (expressed and purified from *E. coli*). After obtaining serum in rabbits, the antibodies were purified by affinity chromatography. The titer of these antibodies was tested by ELISA and the specificity was confirmed by IB with purified recombinant protein. The purified AdACS3 and AdACO3 antibodies were used at 1:5000 dilution.

### Stable genetic transformation in kiwifruit

The full lengths of *AdACO3* and *AdERF105L*/*AdWRKY29* were inserted into the pBTEX-His and pSAK277 vectors downstream of the CaMV35S promoter and then transferred into *A. tumefaciens* EHA105. Corresponding primers are listed in Supplementary Data Table S2. Leaves of plantlets cv. ‘Donghong’ were used for transformation following a modified method [29, 38]. The shoot induction medium and rooting medium contained 50 and 25 mg/L kanamycin for screening, respectively. Positive transgenic plants were identified by RT–qPCR and IB on mRNA and protein levels, respectively.

### Co-immunoprecipitation and co-immunoprecipitation–mass spectrometry

Protein–protein interactions analyzed by CoIP were performed using tobacco leaves expressing the corresponding protein with the HA or Myc tag. The full-length coding sequences of *AdACS3* and *AdAP* were inserted into 1300-3HA vector, and full lengths of *AdACO3* and *AdMPK16* were inserted into 1300-4Myc vector. Sequences of primers for vector construction are listed in Supplementary Data Table S2. The constructs were then transformed into *A. tumefaciens* GV3101 and infiltrated into tobacco leaves (*N. benthamiana*). Two days after infiltration, the samples were collected and frozen in liquid nitrogen for protein extraction. Protein extracts were immunoprecipitated using Anti-Myc Affinity Gel (Yeasen; 20587ES03) with a few left as input. IP proteins together with input proteins were analyzed by immunoblotting with HA or Myc antibody.

Immunoprecipitation of *ACO3* transgenic plants was performed using Protein A Agarose (Beyotime; P2051). A total of 200 μL of extracted protein was incubated with AdACO3-specific rabbit polyclonal antibody or rabbit normal IgG (Beyotime; A7016) at 4°C overnight, then 40 μL of agarose beads were added and the preparation was incubated at 4°C for 3 hours. The suspension was then centrifuged at 2000 g for 5 minutes at 4°C. Beads were collected and washed with 200 μL of extraction buffer five times. Then 40 μL of protein loading buffer was added and the preparation was boiled for 5 minutes. Boiled proteins were separated by SDS–PAGE and gel pieces were destained in 50 mM NH_4_HCO_3_ in 50% acetonitrile. Subsequent in-gel digestion, LC–MS/MS analysis, and data processing were performed by APTBio (Shanghai, China).

### Luciferase complementation imaging and firefly luciferase imaging assay

LCI tests were conducted following the previously described protocol [36]. The full-length coding sequences of *AdACS3*, *AdMPK16*, *AdACO3*, and *AdAP* were constructed into both pCAMBIA1300-nLuc and pCAMBIA1300-cLuc vectors. All the constructs were transformed into *A. tumefaciens* GV3101 and transiently infiltrated into tobacco (*N. benthamiana*) leaves with a ratio of 1:1. For firefly luciferase imaging assay, full-length *AdACS3* was constructed into pBI121-Luc vector while the full-length coding sequences of *AdMPK4*/*16*/*18* were constructed into SK vector. The constructs were transferred into *A. tumefaciens* GV3101, then cultured and dissolved in infiltration buffer and adjusted to an OD_600_ of 1. The prepared cultures of ACS3 and MPK4/16/18 were mixed at a ratio of 1:10, and infiltrated into tobacco leaves (*N. benthamiana*). Two days after infiltration, luciferase activity was determined using NightSHADE LB 985. The primers are listed in Supplementary Data Table S2.

### Statistical analysis

Figures were drawn with GraphPad Prism 7. Phylogenetic tree analysis was conducted with ClustalX and FigTree. The heat map was drawn with TBtools. Data analysis was performed with Microsoft Excel.

## Acknowledgements

We thank the team at the Agricultural Experiment Station of Zhejiang University for plant care. This research was supported by the National Key Research and Development Program (2018YFD1000200), the National Natural Science Foundation of China (32072635), and the Key Research and Development Program of Zhejiang Province (2021C02015), the Fruit New Varieties Breeding Project of Zhejiang Province (2021C02066-8), and the Fok Ying Tung Education Foundation (161028).

## Author contributions

X.-R.Y., J.W., and W.-Q.W. were involved in the experimental design; J.W. performed the experiments with the assistance of W.-Q.W., X.-F.L. and H.-Q.Z.; J.W. wrote the paper; W.-Q.W., A.C.A. and X.-R.Y. revised and commented on the manuscript.

## Data availability

Transcriptome sequencing data are available at NCBI PRJNA730419.

## Conflict of interest

The authors declare no competing interests.

## Supplementary data

Supplementary data is available at *Horticulture Research* online.

## Supplementary Material

Web_Material_uhac116
