# Supplementary material for: Transcriptional and post-transcriptional regulation of ethylene biosynthesis by exogenous acetylsalicylic acid in kiwifruit
Source: Hortic Res. 2022 May 17;9:uhac116. doi: 10.1093/hr/uhac116 (PMC9347011; doi:10.1093/hr/uhac116)
Supplement: Web_Material_uhac116 [file web_material_uhac116.docx]

**Supplementary Figures S1-S8 and Tables S1-S2**

**Article title:** Transcriptional and Post-transcriptional Regulation of Ethylene Biosynthesis by Exogenous Acetylsalicylic Acid in Kiwifruit

**Authors:** Jian Wang, Xiao-fen Liu, Hui-qin Zhang, Andrew C. Allan, Wen-qiu Wang, Xue-ren Yin

**
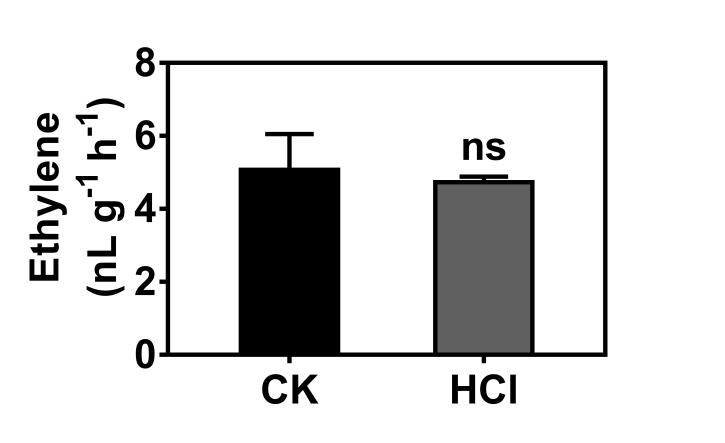
**

**Supplementary Fig. S1. Effects of pH on ethylene production in kiwifruit discs.** Fruits were processed into flesh discs with a diameter of 1 cm and a thickness of 2 mm, then immersed in pH 3.5 hydrochloric acid solution (HCl) and water (CK) at 28oC for 6 h. Ethylene production were measured. Error bars indicate SEs from three replicates. The statistical analysis was performed using two-tailed Student’s *t*-tests. ns represents no significant.


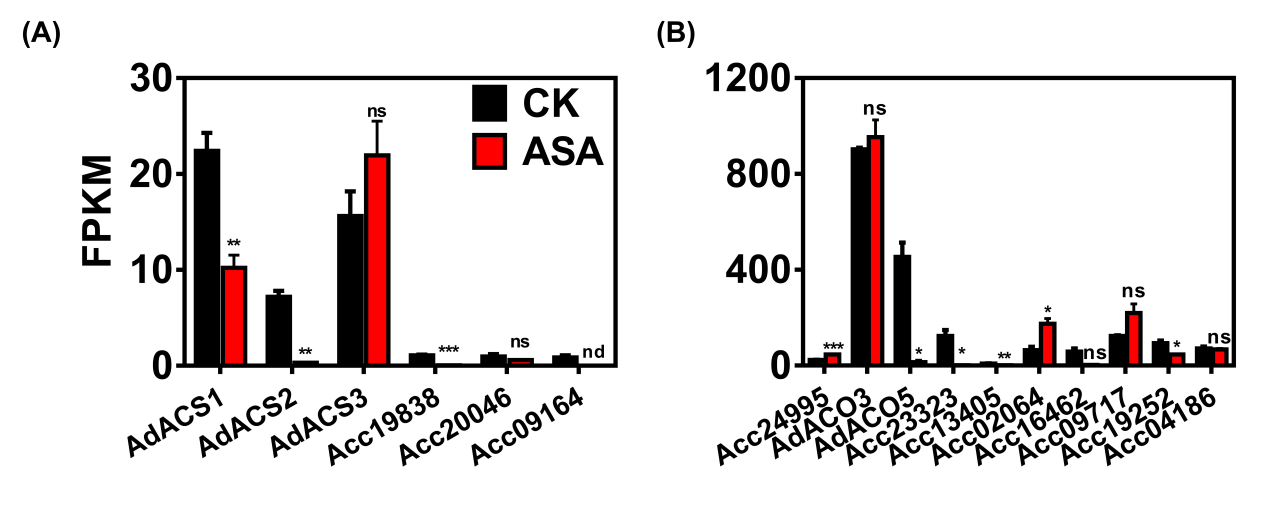


**Supplementary Fig. S2. FPKM values of ACS (A) and ACO (B) gene families in kiwifruit discs.** Fruit discs were treated with 0.5 mM acetylsalicylic acid (ASA) or control (CK) at 28^o^C for 6 h. Error bars indicate SEs from three replicates. The statistical analysis was performed using two-tailed Student’s *t*-tests. The asterisks indicate significant differences: **P* < 0.1; ***P* < 0.01; ****P* < 0.001. ns represents no significant; nd represents not detected.


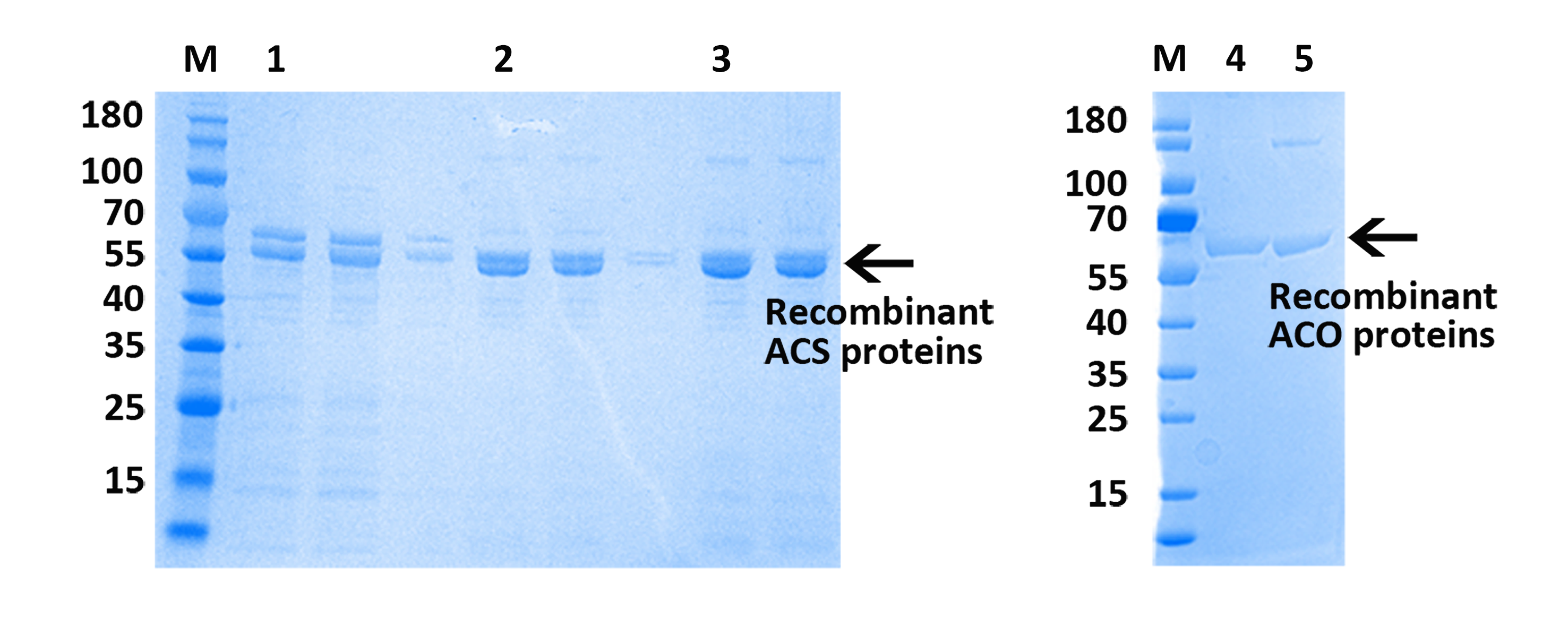


**Supplementary Fig. S3. Electrophoresis and coomassie blue staining of recombinant proteins of five candidate genes.** Recombinant protein purified after expression in *Escherichia coli* strain BL21. Lane 1-5 represent recombinant AdACS1/2/3 and AdACO3/5 protein, respectively. M represents protein marker.

**
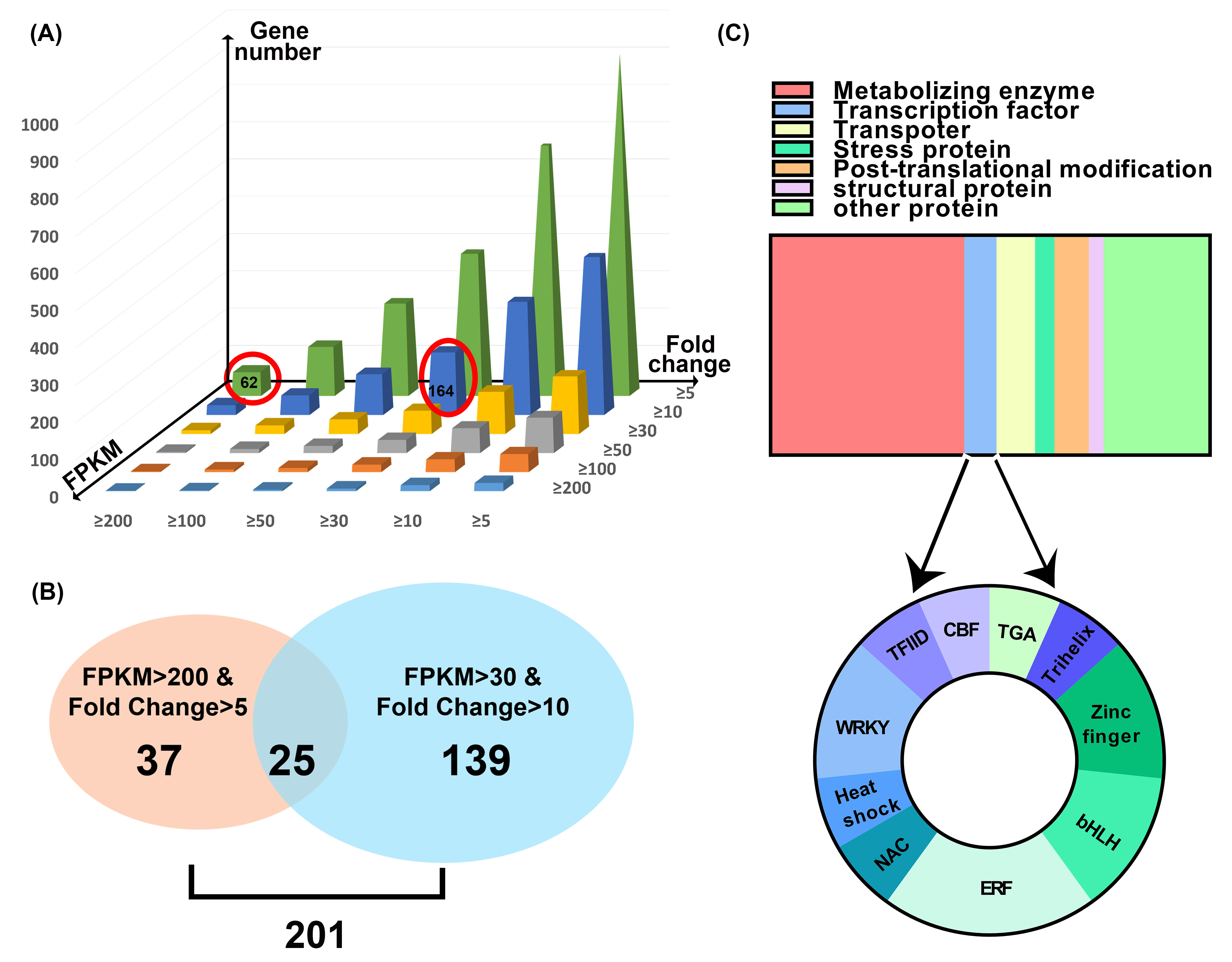
**

**Supplementary Fig. S4. Identification of DEGs responding to ASA treatment by RNA-Seq analysis.** Samples were analyzed with three biological replicates. **(A)** 5059 DEGs were classified into 36 groups based on FPKM value and absolute fold change. X axis, Y axis, Z axis represent FPKM, absolute fold change, gene numbers, respectively. Two selection criteria for screening DEGs were highlighted by red circles and black numbers represent gene quantity under corresponding criteria. **(B)** The Venn diagram showed the number of highly expressed DEGs (Pink oval) and highly differential DEGs (Blue oval), and 201 candidate genes were chosen by taking the summation of these two groups. **(C)** 201 candidate DEGs were divided into 7 groups based on gene function annotation, and 15 differentially expressed transcription factors were separated to verify transcriptional regulatory effects.
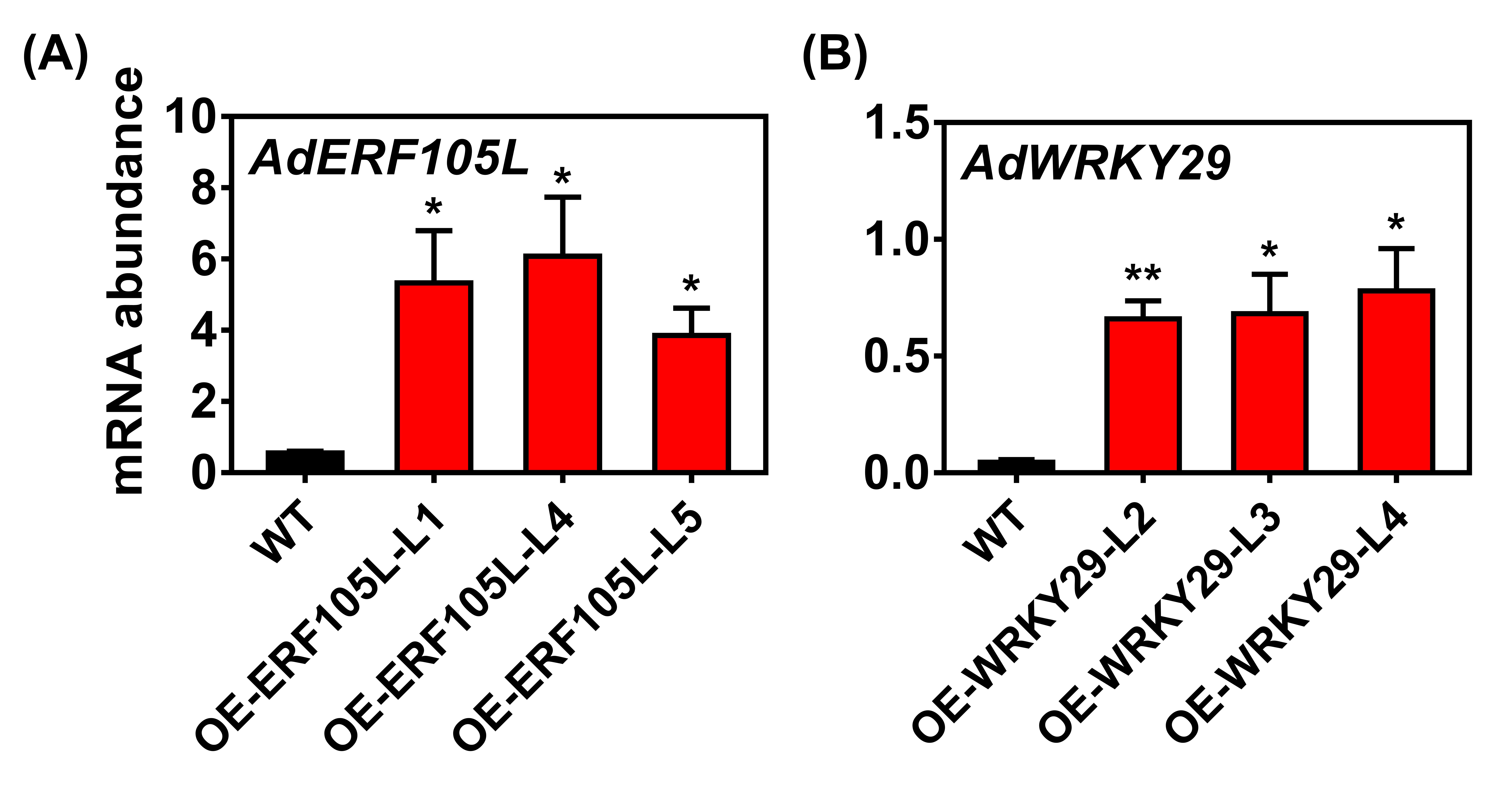


**Supplementary Fig. S5.** **Identification of transgenic kiwifruit plants.** **(A)** Expression of *AdERF105L* in wild type (WT) and *AdERF105L* over-expressed transgenic kiwifruit plants (Line 1/4/5). **(B)** Expression of *AdWRKY29* in WT and *AdWRKY29* over-expressed transgenic kiwifruit plants (Line 2/3/4). Gene expression was analyzed by RT-qPCR and mRNA abundance was the expression relative to that of *AdACT*. Error bars indicate SEs from three replicates. The statistical analysis was performed using two-tailed Student’s *t*-tests. The asterisks indicate significant differences: **P* < 0.1; ***P* < 0.01.

**
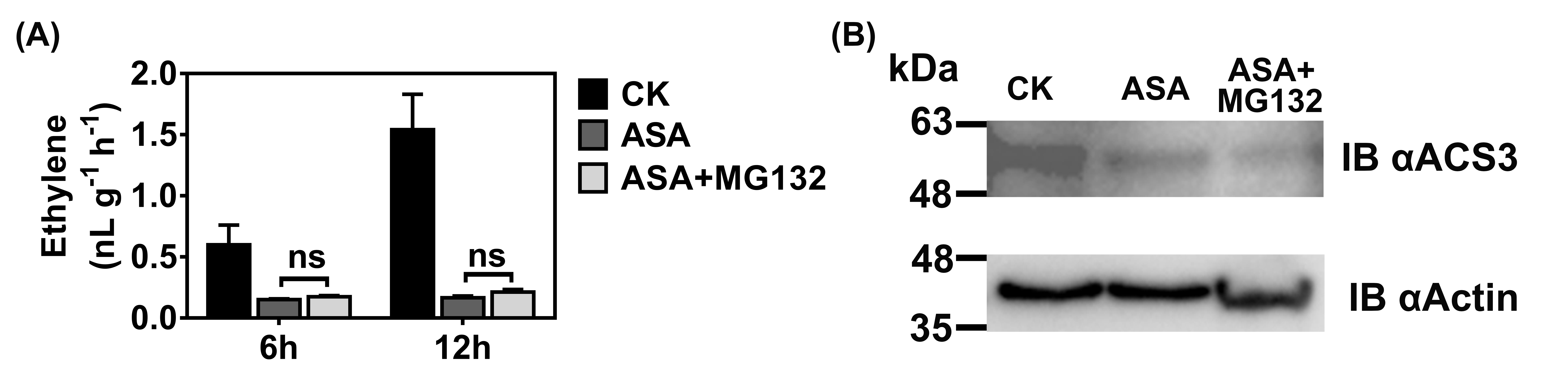
**

**Supplementary Fig. S6. Effetcs of ASA and ASA+MG132 on kiwifruit discs.** **(A)** Fruits were processed into flesh discs with a diameter of 1 cm and a thickness of 2 mm for 0.5 mM acetylsalicylic acid treatments (ASA), 0.5 mM ASA+50 μM MG132 (ubiquitination inhibitors) and control (CK). Fruit discs were incubated at 28^o^C for 6 h and 12 h, respectively. Ethylene production were measured. (B) Protein level of AdACS3 in CK and ASA-treated kiwifruit discs, confirmed by immunoblotting using ACS3 specific antibody. IB with *β*-actin antibody indicate similar loading. Error bars indicate SEs from three replicates. The statistical analysis was performed using two-tailed Student’s *t*-tests. ns represents no significant.


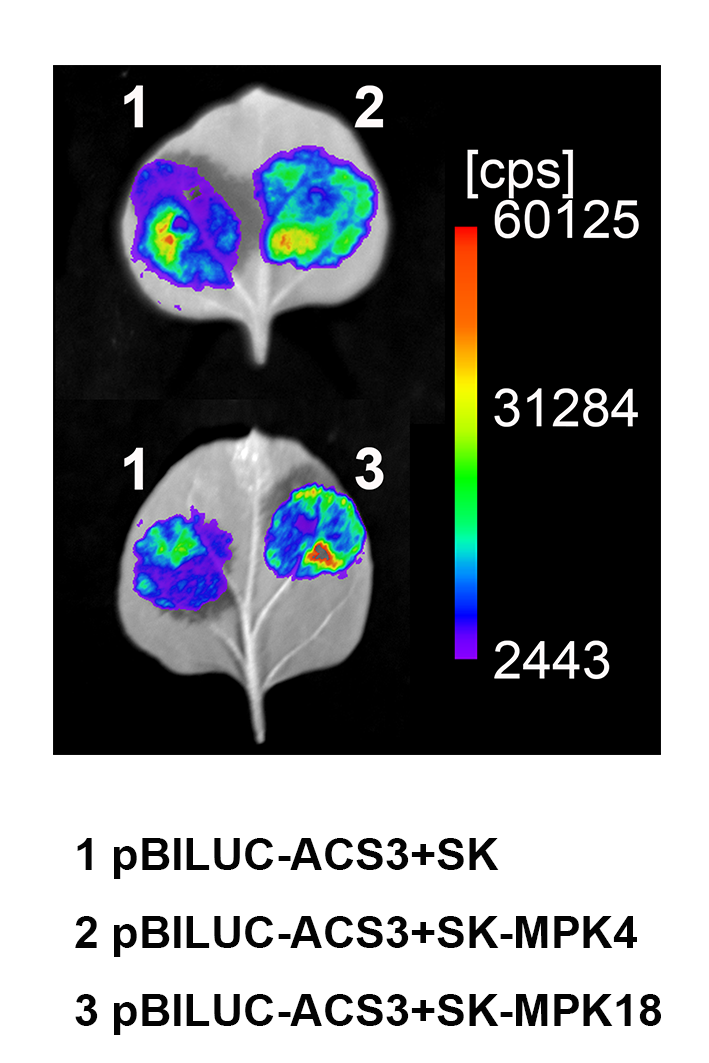


**Supplementary Fig. S7. Firefly luciferase imaging assay of AdACS3 and AdMPK4/18.** AdMPK4/18 were unable to stabilize AdACS3 protein. The strength of LUC signal (showing by different colors) indicate the protein stability of AdACS3.


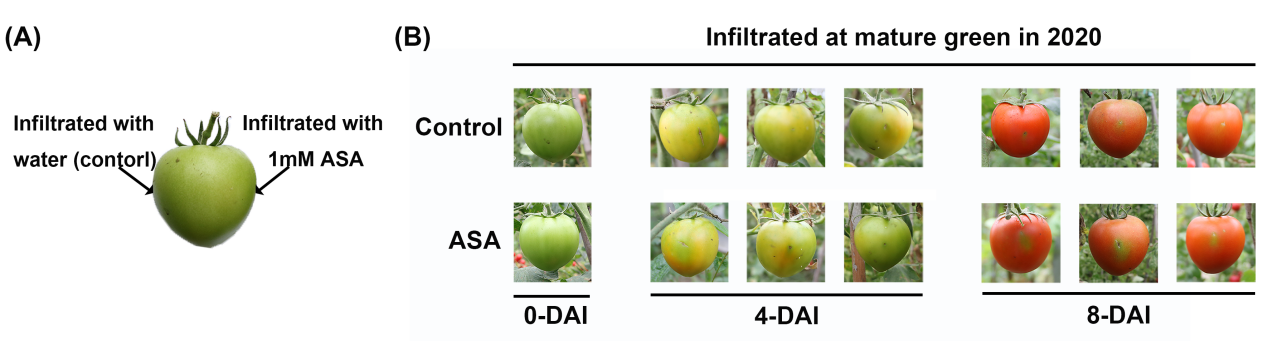


**Supplementary Fig. S8. Effects of ASA on intact mature-green tomato fruits. (A)** Schematic diagram of the infiltration, showing that acetylsalicylic acid (ASA) and water (control) were infiltrated on both sides of the same tomato. Tomato fruits were kept on the branches for subsequent photographing after injection. **(B)** ASA delayed color change around the injection orifices in tomato. DAI, Days after injection.

**Supplementary Table S1. Primers for RT-qPCR.**

| ***Gene*** | ***Primers sequence(5'-3')*** |
| --- | --- |
| *AdACT-F* | TGCATGAGCGATCAAGTTTCAAG |
| *AdACT-R* | TGTCCCATGTCTGGTTGATGACT |
| *AdWRKY29-F* | CTCCACCACCAAAAGACGAAGA |
| *AdWRKY29-R* | TCAAATTCTGGAATCGTAGTAT |
| *AdERF105L-F* | CGTCTTGAACTTCCCTTTGGACG |
| *AdERF105L-R* | GTTAACGAACGTTTCACTACTT |
| *AdACS3-F* | AGGCTAATAATCCACGAGGTGA |
| *AdACS3-R* | CTTGATCCAATCCCTTTCTTTG |
| *AdACO3-F* | CAGGAAGCGAGGCTGTCATCTA |
| *AdACO3-R* | ATGGCCTTCATGGCTTCA |
| *AdMPK16-F* | AGACTTACAGTTGAGGACGCA |
| *AdMPK16-R* | CAGCTCCTTCATCTGTTCCTCG |
| *AdASP-F* | ACGAGCGTGTTTTGGAGCAT |
| *AdASP-R* | CGTCAATCTGATGCCCTCCG |
| *MiACS1-F* | GATTTGAGATGGAGAACAGGAGTTC |
| *MiACS1-R* | GCAAACCCCTTGATTCTGATGTG |
| *MiACO1-F* | GAAGGCGATGAAGGAATTTG |
| *MiACO1-R* | TCCTGGAAGAGCAAGATGATG |
| *DkACS2-F* | GCCGGAGAAGAAGAAGAAGC |
| *DkACS2-R* | AAACGTTAATGGCAGCGCAT |
| *DkACO2-F* | AGCCTCTTTCTACAACCCGG |
| *DkACO2-R* | AGTTAACAGAGGGCTTGGCT |
| *PbACS1-F* | AGACTGAAGGCATTTGTAGGGG |
| *PbACS1-R* | GTGCTCAGACCAGGCTACTTT |
| *PbACO1-F* | CCCCAATGCACCACTCCAT |
| *PbACO1-R* | GGGCGTCCTCAGTTTTCTTCT |
| *SlACS2-F* | GGAGGTTCGTAGGTGTTGAG |
| *SlACS2-R* | AATGGTGAGGGAGGAATAGG |
| *SlACO1-F* | GGAGGCATCATACTTCTGT |
| *SlACO1-R* | CATCACTTCCTGGATTGTA |

**Supplementary Table S2. Primers for vector construction.**

| ***No.*** | ***Vector*** | ***Gene*** | ***Primer Sequence 5'-3'*** |
| --- | --- | --- | --- |
| 1 | pET32a | *AdACS1-F* | ATCGGATCCGAATTCATGAAGCTGCTATCTAGAAA |
| 2 | pET32a | *AdACS1-R* | CGCAAGCTTGTCGACTCGTTCGTCGGGTTCGCGCC |
| 3 | pET32a | *AdACS2-F* | ATCGGATCCGAATTCATGAAGCTGCTCTCTAGAAA |
| 4 | pET32a | *AdACS2-R* | CGCAAGCTTGTCGACTCGTTCTTCGGGCTCGCGTC |
| 5 | pET32a | *AdACS3-F* | ATCGGATCCGAATTCATGGGGTTCATTTCAAGCAA |
| 6 | pET32a | *AdACS3-R* | CGCAAGCTTGTCGACATTCCTTGCCCTAACAAGGG |
| 7 | pET32a | *AdACO3-F* | ATCGGATCCGAATTCATGGAGGCTTTCCCAGTCAT |
| 8 | pET32a | *AdACO3-R* | CGCAAGCTTGTCGACAATTGTTGAAATTGGACCCA |
| 9 | pET32a | *AdACO5-F* | ATCGGATCCGAATTCATGGAGACCTTCCCAGTGAT |
| 10 | pET32a | *AdACO5-R* | CGCAAGCTTGTCGACAGCTGTTGCAATAGGGCCTG |
| 11 | SK | *AdERF105L-F* | GCCCAAGCTGAGCTCATGACGACAGACGAGTCCTT |
| 12 | SK | *AdERF105L-R* | CAGCCCGGGGGATCCCTACGGAGCCACCCGGTTCC |
| 13 | SK | *AdWRKY29-F* | GCCCAAGCTGAGCTCATGCCTCCTTTTCTCTCTTC |
| 14 | SK | *AdWRKY29-R* | CAGCCCGGGGGATCCTCAGCAAGAAAAGCCACTGT |
| 15 | SK | *Achn035131-F* | GCCCAAGCTGAGCTCATGGACAAAGATTACTTCCT |
| 16 | SK | *Achn035131-R* | CAGCCCGGGGGATCCTCAATTTTCATGTGGGATGT |
| 17 | SK | *Achn047841-F* | GCCCAAGCTGAGCTCATGGCCCAGGCGATCCCAAA |
| 18 | SK | *Achn047841-R* | CAGCCCGGGGGATCCTCAATGACGCGGAGGATCTT |
| 19 | SK | *Achn128751-F* | GCCCAAGCTGAGCTCATGTATGGCGATTCCCGAGC |
| 20 | SK | *Achn128751-R* | CAGCCCGGGGGATCCTCAAGCAGATAGATTTGAGA |
| 21 | SK | *Achn152441-F* | GCCCAAGCTGAGCTCATGGAAGGTCATCACCCCCA |
| 22 | SK | *Achn152441-R* | CAGCCCGGGGGATCCCTACATGTGATCTTCTCTTC |
| 23 | SK | *Achn258381-F* | GCCCAAGCTGAGCTCATGGGGAGTGAAACAGAGCA |
| 24 | SK | *Achn258381-R* | CAGCCCGGGGGATCCTCATATCACCATGTCGACGA |
| 25 | SK | *Achn061011-F* | GCCCAAGCTGAGCTCATGATGATGATGATCGGAGA |
| 26 | SK | *Achn061011-R* | CAGCCCGGGGGATCCTTACCAAATATTAGACCCAC |
| 27 | SK | *Achn042271-F* | GCCCAAGCTGAGCTCATGGCGGATTCAGATAACGA |
| 28 | SK | *Achn042271-R* | CAGCCCGGGGGATCCTTAGCTCCGAGACCCGGGAT |
| 29 | SK | *Achn048661-F* | GCCCAAGCTGAGCTCATGACGAGTCCATTGGAGTT |
| 30 | SK | *Achn048661-R* | CAGCCCGGGGGATCCCTAAAATGGCTTCTCCATGT |
| 31 | SK | *Achn104831-F* | GCCCAAGCTGAGCTCATGGCTCTTGAAGCCCTCAA |
| 32 | SK | *Achn104831-R* | CAGCCCGGGGGATCCTTAAATTTGGGGAGGACACA |
| 33 | SK | *Achn157941-F* | GCCCAAGCTGAGCTCATGGCTAACCCTTCTAACGT |
| 34 | SK | *Achn157941-R* | CAGCCCGGGGGATCCTCATCGATAACTCCATAGAT |
| 35 | SK | *Achn247541-F* | GCCCAAGCTGAGCTCATGGAAGGTGTGACAGTTAA |
| 36 | SK | *Achn247541-R* | CAGCCCGGGGGATCCTCAGCCAAACAAGTCATCAG |
| 37 | SK | *Achn287671-F* | GCCCAAGCTGAGCTCATGGACTCCTCCTCCCCGGA |
| 38 | SK | *Achn287671-R* | CAGCCCGGGGGATCCTCAAAATTCAAATTCAAATA |
| 39 | SK | *Achn320041-F* | GCCCAAGCTGAGCTCATGAGCATTGTGCCTAAGGA |
| 40 | SK | *Achn320041-R* | CAGCCCGGGGGATCCTTACACAAACACAGACATCT |
| 41 | LUC | *AdACS1-F* | TCCACTAGTTCTAGAGCGGCCGCACATCAGGGTTGGTCAGTGT |
| 42 | LUC | *AdACS1-R* | TGTTTTTGGCGTCTTCCATGGTTGCAATTTAGGCCCAAAATCC |
| 43 | LUC | *AdACS2-F* | CGGTATCGATAAGCTTAATCTTCTCAGTAATAAGTCCCA |
| 44 | LUC | *AdACS2-R* | TTGGCGTCTTCCATGGTTTTGGAAATCCAGAGAAAACAG |
| 45 | LUC | *AdACO5-F* | CGGTATCGATAAGCTTCGACGGCCCGGGCTGGTATTA |
| 46 | LUC | *AdACO5-R* | TTGGCGTCTTCCATGGTGCTCTCTCTCTCTCTCTCTTAAA |
| 47 | pGEX4t-1 | *AdERF105L-F* | GTTCCGCGTGGATCCATGACGACAGACGAGTCCTT |
| 48 | pGEX4t-1 | *AdERF105L-R* | TCGACCCGGGAATTCCGGAGCCACCCGGTTCC |
| 49 | pGEX4t-1 | *AdWRKY29-F* | GTTCCGCGTGGATCCATGCCTCCTTTTCTCTCTTC |
| 50 | pGEX4t-1 | *AdWRKY29-R* | TCGACCCGGGAATTCGCAAGAAAAGCCACTGT |
| 51 | 2YN/2YC | *AdACS3-F* | TACGAACGATAGTTAATTAATATGGGGTTCATTTCAAGC |
| 52 | 2YN/2YC | *AdACS3-R* | TCCTCCACTAGTGGCGCGCCCATTCCTTGCCCTAACAAG |
| 53 | 2YN/2YC | *AdMPK16-F* | TACGAACGATAGTTAATTAATATGGACACATCGGCTG |
| 54 | 2YN/2YC | *AdMPK16-R* | TCCTCCACTAGTGGCGCGCCCCATGTGCATGTACTCGG |
| 55 | 2YN/2YC | *AdMPK4-F* | TACGAACGATAGTTAATTAATATGTCTGTGGATTCGAGCACTGCTT |
| 56 | 2YN/2YC | *AdMPK4-R* | TCCTCCACTAGTGGCGCGCCCATGAGTGGGAGGGTCAGGGTTGAAT |
| 57 | 2YN/2YC | *AdMPK17-F* | TACGAACGATAGTTAATTAATATGCAGCAAGATCACCGAAAGAAGA |
| 58 | 2YN/2YC | *AdMPK17-R* | TCCTCCACTAGTGGCGCGCCCATACATTCTGGACAAGCCATATTGA |
| 59 | 2YN/2YC | *AdMPK18-F* | TACGAACGATAGTTAATTAATATGGAAAGATACAAAGTTTTGGAGG |
| 60 | 2YN/2YC | *AdMPK18-R* | TCCTCCACTAGTGGCGCGCCCATGACTCTGCTGAAAGGGGGAGGAC |
| 61 | pBILUC | *AdACS3-F* | GAGAACACGGGGGACTCTAGAATGGGGTTCATTTCAAGC |
| 62 | pBILUC | *AdACS3-R* | GGACTGACCACCCGGGGATCCATTCCTTGCCCTAACAA |
| 63 | SK | *AdMPK4-F* | GCCCAAGCTGAGCTCATGTCTGTGGATTCGAGCACTG |
| 64 | SK | *AdMPK4-R* | CAGCCCGGGGGATCCTTAATGAGTGGGAGGGTCAGGG |
| 65 | SK | *AdMPK16-F* | CCCCCGGGCTGCAGGAATTCATGGACACATCGGCTGAGC |
| 66 | SK | *AdMPK16-R* | GGCCCCCCCTCGAGGTCGACTCACATGTGCATGTACTC |
| 67 | SK | *AdMPK17-F* | TACGAACGATAGTTAATTAATATGCAGCAAGATCACCGAAAGAAGA |
| 68 | SK | *AdMPK17-R* | TCCTCCACTAGTGGCGCGCCCATACATTCTGGACAAGCCATATTGA |
| 69 | SK | *AdMPK18-F* | GCCCAAGCTGAGCTCATGGAAAGATACAAAGTTTTGG |
| 70 | nLUC | *AdACS3-F* | ACGGGGGACGAGCTCGGTACCATGGGGTTCATTTCAAGC |
| 71 | nLUC | *AdACS3-R* | CGCGTACGAGATCTGGTCGACATTCCTTGCCCTAACAAG |
| 72 | cLUC | *AdACS3-F* | TACGCGTCCCGGGGCGGTACCATGGGGTTCATTTCAAGC |
| 73 | cLUC | *AdACS3-R* | ACGAAAGCTCTGCAGGTCGACCTAATTCCTTGCCCTAAC |
| 74 | nLUC | *AdMPK16-F* | ACGGGGGACGAGCTCGGTACCATGGACACATCGGCTGAGCACT |
| 75 | nLUC | *AdMPK16-R* | CGCGTACGAGATCTGGTCGACCATGTGCATGTACTCGGGGTTA |
| 76 | cLUC | *AdMPK16-F* | TACGCGTCCCGGGGCGGTACCATGGACACATCGGCTGAGCACT |
| 77 | cLUC | *AdMPK16-R* | ACGAAAGCTCTGCAGGTCGACTCACATGTGCATGTACTCGGGG |
| 78 | SK | *AdMPK18-R* | CAGCCCGGGGGATCCTCAATGACTCTGCTGAAAGGGG |
| 79 | 3HA | *AdACS3-F1* | GGACTCTAGAGGATCCATGGGGTTCATTTCAAGC |
| 80 | 3HA | *AdACS3-R1* | CATCATAAGGATACATGTACGTAACATTCCTTGCCCTAACAA |
| 81 | 3HA | *AdACS3-F2* | CTCCCCAATTTCTTCACCCCTTGTTAGGGCAAGGAATGTTACGTACATGTATC |
| 82 | 3HA | *AdACS3-R2* | GGAAATTCGAGCTCAGGCCCTTAAAGACTAGC |
| 83 | 4myc | *AdMPK16-F1* | GGACTCTAGAGGATCCATGGACACATCGGCTGAGCACT |
| 84 | 4myc | *AdMPK16-R1* | CCATCTTGTCCATGTGCATGTACTCGG |
| 85 | 4myc | *AdMPK16-F2* | CCGAGTACATGCACATGGACAAGATGGAGCAAAAGCTCAT |
| 86 | 4myc | *AdMPK16-R2* | CGATCGGGGAAATTCGAGCTCTCTAGTCTAGCCCAAGTCCTCTTCA |
| 87 | RSFDuet | *AdACS3-F* | AGCCAGGATCCGAATTCTATGGGGTTCATTTCAAGC |
| 88 | RSFDuet | *AdACS3-R* | TTATGCGGCCGCAAGCTTCTAATTCCTTGCCCTAACAA |
| 89 | pET32a | *AdMPK16-F* | ATCGGATCCGAATTCATGGACACATCGGCTGAGCACT |
| 90 | pET32a | *AdMPK16-R* | CGCAAGCTTGTCGACCATGTGCATGTACTCGGGGAAT |
| 91 | pBTEX | *AdACO3-F* | GGACAGGGTACCCGGGGATCCATGGAGGCTTTCCCAGTC |
| 92 | pBTEX | *AdACO3-R* | AGGGCATGCCTGCAGGTCGACTTAGTGGTGGTGGTGGTGGTGAATTGTTGAAATTGG |
| 93 | nLUC | *AdACO3-F* | ACGGGGGACGAGCTCGGTACCATGGAGGCTTTCCCAGTC |
| 94 | nLUC | *AdACO3-R* | CGCGTACGAGATCTGGTCGACAATTGTTGAAATTGGACC |
| 95 | cLUC | *AdACO3-F* | TACGCGTCCCGGGGCGGTACCATGGAGGCTTTCCCAGTC |
| 96 | cLUC | *AdACO3-R* | ACGAAAGCTCTGCAGGTCGACTCAAATTGTTGAAATTGG |
| 97 | nLUC | *AdASP-F* | ACGGGGGACGAGCTCGGTACCATGGCTTCCACTTCTCAT |
| 98 | nLUC | *AdASP-R* | CGCGTACGAGATCTGGTCGACATCAATTATGGTGAGGTT |
| 99 | cLUC | *AdASP-F* | TACGCGTCCCGGGGCGGTACCATGGCTTCCACTTCTCAT |
| 100 | cLUC | *AdASP-R* | ACGAAAGCTCTGCAGGTCGACCTAATCAATTATGGTGAG |
| 101 | 3HA | *AdASP-F1* | GGACTCTAGAGGATCCATGGCTTCCACTTCTCATC |
| 102 | 3HA | *AdASP-R1* | CATCATAAGGATACATGTACGTAACATCAATTATGGTGAGGTTGAAG |
| 103 | 3HA | *AdASP-F2* | CTTCAACCTCACCATAATTGATGTTACGTACATGTATC |
| 104 | 3HA | *AdASP-R2* | GGAAATTCGAGCTCAGGCCCTTAAAGACTAGC |
| 105 | 4myc | *AdACO3-F1* | GGACTCTAGAGGATCCATGGAGGCTTTCCCAGTC |
| 106 | 4myc | *AdACO3-R1* | CCATCTTGTCAATTGTTGAAATTGGACCC |
| 107 | 4myc | *AdACO3-F2* | GGGTCCAATTTCAACAATTGACAAGATGGAGCAAAAGCTCAT |
| 108 | 4myc | *AdACO3-R2* | CGATCGGGGAAATTCGAGCTCTCTAGTCTAGCCCAAGTCCTCTTCA |
| 109 | pSAK277 | *AdACO3-F* | ACTAGTGGATCCAAAGAATTCATGGAGGCTTTCCCAGTC |
| 110 | pSAK277 | *AdACO3-R* | TCATTAAAGCAGGACTCTAGATCAAATTGTTGAAATTGGA |
| 111 | pSAK277 | *AdASP-F* | ACTAGTGGATCCAAAGAATTCATGGCTTCCACTTCTCATC |
| 112 | pSAK277 | *AdASP-R* | TCATTAAAGCAGGACTCTAGACTAATCAATTATGGTGAGGTTG |
| 113 | pBTEX-His | *AdACO3-F* | GGACAGGGTACCCGGGGATCCATGGAGGCTTTCCCAGTC |
| 114 | pBTEX-His | *AdACO3-R* | AGGGCATGCCTGCAGGTCGACTTAGTGGTGGTGGTGGTGGTGAATTGTTGAAATTGG |
